# Multidisciplinary integrated Parent and Child Centres in Amsterdam: a qualitative study

**DOI:** 10.5334/ijic.887

**Published:** 2013-04-12

**Authors:** Vincent Busch, Henk François Van Stel, Johannes Rob Josephus De Leeuw, Edward Melhuish, Augustinus Jacobus Petrus Schrijvers

**Affiliations:** Julius Center for Health Sciences and Primary Care, University Medical Center Utrecht, Utrecht, The Netherlands; Julius Center for Health Sciences and Primary Care, University Medical Center Utrecht, Utrecht, The Netherlands; Julius Center for Health Sciences and Primary Care, University Medical Center Utrecht, Utrecht, The Netherlands; Institute for the Study of Children, Families and Social Issues, Birkbeck University of London, London, UK; Julius Center for Health Sciences and Primary Care, University Medical Center Utrecht, Utrecht, The Netherlands

**Keywords:** child health, family centres, integrated services, multidisciplinary cooperation, health professionals, Netherlands

## Abstract

**Background:**

In several countries centres for the integrated delivery of services to the parent and child have been established. In the Netherlands family health care service centres, called Parent and Child Centres (PCCs) involve multidisciplinary teams. Here doctors, nurses, midwives, maternity help professionals and educationists are integrated into multidisciplinary teams in neighbourhood-based centres. To date there has been little research on the implementation of service delivery in these centres.

**Study design:**

A SWOT analysis was performed by use of triangulation data; this took place by integrating all relevant published documents on the origin and organization of the PCCs and the results from interviews with PCC experts and with PCC professionals (n=91). Structured interviews were performed with PCC-professionals [health care professionals (n=67) and PCC managers n=12)] and PCC-experts (n=12) in Amsterdam and qualitatively analysed thematically. The interview themes were based on a pre-set list of codes, derived from a prior documentation study and a focus group with PCC experts.

**Results:**

Perceived advantages of PCCs were more continuity of care, shorter communication lines, low-threshold contact between professionals and promising future perspectives. Perceived challenges included the absence of uniform multidisciplinary guidelines, delays in communication with hospitals and midwives, inappropriate accommodation for effective professional integration, differing expectations regarding the PCC-manager role among PCC-partners and the danger of professionals’ needs dominating clients’ needs.

**Conclusions:**

Professionals perceive PCCs as a promising development in the integration of services. Remaining challenges involved improvements at the managerial and organizational level. Quantitative research into the improvements in quality of care and child health is recommended.

## Introduction

Parent and Child Centres (PCCs) are an integrated care innovation in Amsterdam, the Netherlands designed to support better parenting, to strengthen parenting competencies, to identify social and health risks at an early stage and to offer early interventions in case of problems with developments or parenting of children. The PCCs are the first contact that new parents in the Netherlands have with the supporting health and social care system. In the PCCs they get into contact with the health and social care services of youth health nurses and—doctors, general practitioners midwives and maternity help professionals. Services such as regular health check-ups, midwife consultations, parenting advice and the child receiving proper vaccinations are integrated in, and coordinated from, the PCC. In short, PCCs perform a *gatekeeper function* in the Amsterdam health and social care system; through them new parents that need and/or want support in any form in relation to parenthood, medical and psychosocial care advice and family affairs in general, ideally, get identified and facilitated with proper help.

During the last decade similar innovations emerged in other regions of the country, but also in different countries, such as England, Germany, Belgium and Finland [1–3]. Amsterdam is the first city in the Netherlands with long standing experience with integrated youth health care and it started with its PCC in 1997. Before that time, the Amsterdam youth health and social care system was characterised by its fragmentized organization. Parents often did not know where to go to for advice regarding parenting issues and services often did not match their needs. This resulted in the increased use of various specialized secondary health care services, despite the relatively low prevalence of complex care cases [4]. Proper support services that are both multidisciplinary in nature and universally available at a local level, might prevent premature referrals and the according intensive and expensive (curative) care [5–13].

The Amsterdam PCCs offer just that, namely integrated, multidisciplinary services that are easily accessed, based in a community setting for (would-be) parents and children [14]. Each of the 14 municipality districts of Amsterdam has at least one working PCC. The PCCs offer general advice and parenting support as well as tailored help, specialized referrals to secondary care services, consultations with special education and with general practitioners [15]. They were developed bottom-up by professionals and evolved into a city-wide system change in multidisciplinary care and collaboration [16, 17].

**Figure 1.**
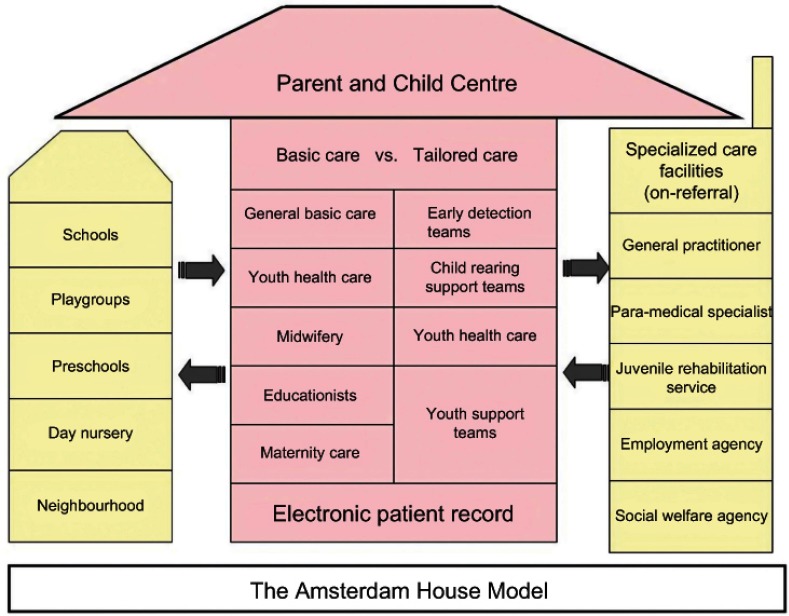
The Amsterdam House Model.

PCCs also facilitate partnerships with other agencies such as with the provincial Youth Care Agency, with the School Care and Advice Teams and many others [1, 2, 18]. For the full scope of these partnerships, see Figure 1. In this new organizational structure Amsterdam’s professionals and managers often refer to the PCC as the spider in the web of information, care, and early identification of problems and professional referrals.

The development and functioning of the Amsterdam PCCs has not yet been described in the current literature, nor have the various developments and consequences been evaluated. PCCs in Amsterdam are a structural innovation with the objective to enhance inter-professional partnership. Due to their pioneering role in the Netherlands, Amsterdam’s PCC were chosen as a case study for the qualitative evaluation of inter-professional partnership by means of a focussed (Strengths, Weaknesses, Opportunities and Threats) SWOT analysis [19]. This article addresses the question: What are the perceived advantages and barriers in inter-professional partnership from the points of view of the PCC professionals and experts in comparison to the previous system of youth health services in Amsterdam? In the current study the definitions of Butt for *inter-professional* and for *partnership* are used [20]. Based on a literature study Butt defines three groups of characteristics influencing inter-professional partnership: 1. Salient attributes (agreement between professionals about the collaboration, collegial relationships, interdependency and leadership), 2. Organizational factors (structure, culture, administrative support, resources, coordination and communication mechanisms, sustainability and clinical guidelines) 3. Systemic factors (e.g., differences in social status between professionals, professional regulations, individualism and autonomy feelings of professionals, lack of knowledge in professionals and financial incentives for different professionals). Butt et al. [20] distinguishe three types of outcome: 1. Partnership functioning (this is improvement in factor 1); 2. System capacity (this is improvement in factors 2 and 3); and 3. Individual and population health outcomes (this is the final outcome). Butt et al. [20] developed not only a conceptual model but also tools to measure their model quantitatively. However, because of limited resources a qualitative study design via a focussed SWOT analysis was chosen. The main purpose of the current study is to measure perceptions of the effects of the creation of PCCs on the salient attributes which are mentioned above. In the discussion paragraph we refer to Butt when embedding our findings in a theoretical framework and we then compare our findings with other research on inter-professional partnership and collaboration.

## Methods

### Study design

This qualitative study, based on grounded theory [21], was set up as a two-layered analysis on the strengths, weaknesses, opportunities and threats (SWOT-analysis) of the current and future development of PCCs in Amsterdam. For the first ‘professional’ layer, professionals working in or with a PCC were interviewed to evaluate daily practice in PCCs. For the second ‘expert’ layer, people who were involved with the development of PCC and/or similar developments in other parts of the Netherlands were interviewed to evaluate PCCs from a policy viewpoint. Additionally, a review on local (policy) documentation on PCCs was performed to describe the PCC background and interpret interview findings. Triangulation of the different results is done in the discussion.

### Documentation study

The documents used in the documentation study were provided by the main organizations that were involved with the organization and origin of the PCCs; they entailed all relevant documents with regard to the topic. These documents were used to describe the PCC background and to help interpret interview findings. Organizations that provided documents included the municipality of Amsterdam, the municipal health service, and a local primary care organisation.

### Interviews

Structured interviews with open questions on a pre-set topic list were held with various PCC professionals (67 health care professionals, 12 managers) and 12 PCC experts. The interviewed professionals were pre-selected by the organizations in which they worked. They were requested to be interviewed, if they had: 1. At least 5 years of experience in their current profession in Amsterdam; and 2. Experience in both a period without PCCs and within a PCC-setting. PCC professionals and managers of all disciplines were chosen with as much variety as possible. The 79 interviewees included youth health nurses (18), youth health doctors (12) general practitioners (8), professionals outside the PCC to whom PCC-professionals refer (e.g., paediatricians) (7), educationists (7), midwives and maternity help professionals (7), youth physiotherapists, dieticians and speech therapists (7) and managers of PCCs (12). The respondents had an average age of 46 years (range 28 to 61 years, left-skewed) and an average of 17 years of working experience in the field in Amsterdam (range 5–40 years, left-skewed). To achieve a representative pool of professionals 33 out of the 79 interviewed professionals originated from the ‘inner layer’ of the Amsterdam House Model (Figure 1, centre); whose main work base of operations is (literally) within one or more PCCs. The other 46 professionals mainly worked from outside of the PCC, but in close contact with the PCC; these professionals were defined to be from the ‘outside layer’ of the Amsterdam House Model (Figure 1, outside bases).

The 33 ‘inner layer’ professionals worked in 20 different PCCs throughout Amsterdam. The other 46 professionals, the ‘outside layer’ professionals, primarily worked elsewhere, but had (strong) working relations with one or more PCCs and/or worked at one or more PCCs part-time.

The PCC experts were people who were involved with the development of PCC (and similar developments in other parts of the Netherlands) and were recommended by the main PCC-related organizations and the research team. These experts provided a more in-depth embedding into similar developments in other parts of the country and into the international academic literature. Therefore, they provided a valuable contribution to the triangulation process to improve data quality and reliability.

The professional’s interviews themes all related to the PCC-introduction and consisted of 1) what changed in their professional practice, 2) if and how their multidisciplinary relations were affected, 3) what were the strong and weak aspects of the current multidisciplinary relation, and 4) what they identified as possible future opportunities and/or threats for those multidisciplinary relations and the content of their professional practice in the current PCC-setup.

The expert interviews focussed on the same themes as the interviews with field professionals, with the addition of several topics that were not discussed in detail with the professionals. These topics included: the position of parent and child within the PCC and the role that the different professional disciplines should have within the PCCs; the financing structure of the PCCs and core professionals’ practices; what the method of record keeping for client data should be; how certain privacy regulations should be handled; and finally how the PCCs should be housed.

### Interview analyses

The interviews were written out verbatim and checked for factuals and inconsistencies with the documentation study. This enabled triangulation of information from the both types of interviews and documents [22]. Afterwards, the interview transcripts were sent to the interviewees to allow them to provide the researchers with possible corrections and additional information (member checking), prior to the coding and analyses. If the researchers still came across contradictions, the interviewers contacted the respondent for further clarification as an additional measure of checking data reliability.

All interviews were coded with the qualitative data-analysis program NVIVO 8 [23] and analysed by means of a SWOT analysis. The interview coding scheme was based upon a topic list derived from the documentation study and an expert focus group, prior to the current study. Both descriptive and analytic codes were used. Any new topics that emerged during the qualitative analysis were checked in all interviews (constant comparison or ‘axial coding’). This provided the necessary input for the interviews to perform a focussed SWOT analysis. Eventually, all three forms of data collection (documentation study, expert interviews and interviews with professionals) were all integrated into one synthesis in the Discussion section. The quotes and findings in the results section comprise of information that was gathered from both the interviews with professionals and those with experts.

To maintain confidentiality, respondents were anonymous by coding them 1 to 79. Results were interpreted independently by three researchers.

## Results

There appeared to be consistency in the answers of the professionals and managers. Five themes were derived from the interview questions. The results will be structured according to these themes. The derived themes constitute: 1. The existing regular collaborations between various disciplines in PCCs 2. The influence of PCCs on multidisciplinary communication 3. The strengths and challenges of working under one roof in a PCC 4. The strength and challenges of the position of PPC-managers, and 5. The future strength and challenges for the multidisciplinary relations. Table 1 summarizes the main positive and negative remarks made per item.

### The regular collaboration between various disciplines

The professions of youth health nurses, youth health doctors, educationists, midwives and maternity help professionals together form the basic core of the PCCs (Figure 1). Although being part of the same multidisciplinary team (see Figure 1), midwives and maternity help professionals do not operate from the same building as the other core partners. They operate from private, independent organizations often working from the client’s home. Thus, they are not specifically bound to a certain municipality district or PCC and are therefore often affiliated with multiple PCCs [1, 2], whereas some are not affiliated with any PCC at all; this is not a compulsory organizational feature for them. The former well-baby clinics were chosen as the PCCs’ base of operations, since they were already a low-threshold place for families to visit regarding issues concerning health, child development and parenting [15]. They offered parents support and education through pregnancy, childbirth and the postnatal period. The familiarity with the well-baby clinics in combination with the high outreach of the Amsterdam Municipal Health Service (92–98% of all children between ages 0 and 4 [24]) make the PCC an easily accessible place for parents to visit. Respondents formulated this as follows:

“The PCC is a much easier accessible place for clients than GP’s and doctor’s practices were in the past. When children used to be referred they would often never get to their designated health professional; about a quarter of the clients would fall into this category. Nowadays, this group of non-arrivals is almost zero, due to the PCC being an easy accessible, familiar place for children and families” (#51).

Respondents perceived that PCCs increase the continuity of care. The following quotes illustrate this perception:

“The threshold to come and visit us has become lower for parents now that we’re all in the same building. Therefore, clients tend to disappear from our radar less quickly” (#47).

Added to this perceived improvement in continuity respondents noted a better client accessibility. Professionals also noted better collaboration, because, as it was stated, working from the same building/workplace leads to more smooth and frequent inter-professional contact. Professionals were now able to link a name to a person, a face and a profession of colleagues.

However, challenges still remained, i.e., professionals feel that they are not (yet) supported with a uniform set of multidisciplinary protocols and work procedures for their daily practices. Despite the positive attitude of professionals towards the new, multidisciplinary practice, critiques were expressed in relation to the organizational structures not being adapted to the multidisciplinary functional practices yet. One of the Interviewees formulated it as follows:

“Many professionals still process client/patient information through their own standardized procedures, which have not yet been fine-tuned to the extensive multidisciplinary collaborations as are in effect in the new PCC-setting. This adds to the bureaucracy and inefficiency of information processing” (#19).

Interviewees indicated a need for a front desk employee in the PCC to refer clients to the appropriate service(s) within the PCC. PCCs function as a gatekeeper of the specialized health care system. Therefore, a proper triage referral system from primary to secondary health care is needed. However, due to uncertainties regarding required competencies and expected job specifications for such a front desk employee this is still lacking.

Professionals also noted that standardized procedures to collect and store information are not properly fine-tuned, do not fit into existing privacy regulations and often do not have active feedback moments integrated into the new multidisciplinary communication lines. Some quotes illustrate these remarks made by many professionals:

“There is a strong need from the professionals on to adjust current privacy regulations to the novel, multidisciplinary work situation. This forces professionals to take their appropriate responsibilities and it makes multidisciplinary work more effective” (#3).

### Influence on multidisciplinary communication

Respondents were enthusiastic about the achieved improvements with regard to multidisciplinary, professional communication that developed since the development of the PCCs:

“The communication is going more smoothly nowadays. There are strong communication lines between professions in this new work situation. It provides a feeling of enthusiasm and easy accessibility when you know which face belongs to which name and profession.” (#18).

However, hesitations and challenges were also indicated regarding the communication between hospitals and PCCs and between midwives and other professionals. However, this was indicated to be more related to practical examples of issues that were not yet working smoothly instead of critiques on the systemic changes that the PCCs represent.

### Strengths and challenges of working under one roof

A typical comment made by an interviewee is presented here below. It notes the appreciated advantage of contacting other professionals more easily:

“Everybody is present in the same building nowadays, which makes getting into contact with them easier; you do not have to schedule entire meetings for every little thing” (#26).

Similar to the situation concerning the multidisciplinary communications the practical organization of the actual joint facilities is also still a work in progress. For example some respondents indicated that the building requirements are not in line with the pursued goals of the agreed upon collaborations. Issues such as the lack of sufficient meeting rooms; having split-up work places; or dealing with financial issues were provided as examples of this.

“We have been informed that accommodations possibly have to be financed by the professionals and their organization. If that would become the situation many professionals would have to be forced to abandon PCC efforts” (#67).

### Strengths and challenges of the position of the PCC-managers

Respondents were positive about the availability of a PCC-Manager. One respondent explained:

“The PCC-manager is provided with the power, responsibility and opportunity to speed up collaborating efforts between different disciplines. This is an important, positive development for professionals in the field” (#45).

Respondents even want an expansion of the PCC manager’s tasks:

“Currently, the responsibility for total case-management is often dropped off at the individual professional, while this exceeds the range of their capacity and competencies. The individual professional has neither the time nor the money to take this on properly” (#6). “A PCC-manager’s function can prove vital in such situations” (#45).

Interviewees also noted some challenges concerning the fulfilment of the function PCC-manager, mostly due to differing expectations. One respondent said:

“The problem for the PCC manager is that in every care providing organization and municipal agency the vision on what a PCC-manager function entails differs. Some stated that in their district the PCC-manager is merely the person that updates the professionals on new developments, while in other districts the PCC-manager is the person that all professionals answer to as their chief. A more clearly defined, uniformly accepted role is needed for the PCC-manager” (#10).

### Future opportunities and threats of PCCs

Interviewees were optimistic when it regarded the future of the PCCs. It was stated that the concept of multidisciplinary, more effective health care providing practices is promising, as long as certain underlying issues are tackled in order for the PCCs to become sufficiently effective practices. Mentioned issues such as improving organizational structures, defining roles and competency profiles for different professionals and organize multidisciplinary meetings in more structured fashion were stated as important issues to tackle in this respect.

Respondents, especially experts, also noted that the development of organizing care in a client-centred fashion (instead of it remaining professional-centred) as well as the integration of the different health care services has insufficiently taken place in practice. Many of the organizations still think and act as separate, mono-disciplinary entities instead of parts serving a ‘higher structure’ within a multidisciplinary environment. A further integration of services will require changes in competencies and tasks of professionals to better suit the needs of the client and integrated care processes. Some respondents are afraid of a development in which PCCs expand too much, creating once again a fragmentized situation, dominated by mono-disciplinary organizations.

Furthermore, an aspect that the experts indicated to be necessary for proper future inter-professional cooperation was installing a coherent professional payment structure with appropriate protocols and legislations. Currently, midwives and maternity help professionals are not accredited or reimbursed to attend multidisciplinary, casuistic meetings. Therefore they are forced to attend in their own time, while these meetings take place within working hours. Therefore, they are almost always absent during those meetings. Additionally, according to the professionals, their organizations dictate the philosophy that “*the one holding the money is the one with the decisive vote”*. Therefore, the current payment system does not foresee in the conditions for equal partnerships. This issue was indicated by the experts and several professionals as one of the main gaps in the current PCC-organizational structure and a threat to inter-professional cooperation. This issue was illustrated by the following quote from a respondent:

“We as midwives, but this also concerns e.g., physiotherapists and maternity help professionals, do not get paid or accredited for many meetings and PCC-efforts. We attend out of concern for the collaborations, but it’s certainly not cheap to keep this up” (#70).

Also, multidisciplinary protocols and procedures that concern ethical and privacy related legislations are currently either incomplete or lacking. The absence of such protocols significantly hampers the quality of multidisciplinary practices, according to the interviewees, because professionals cannot share important client details with other professionals and it is often unclear who is responsible for the handling of which (client) information.

## Discussion

The results indicated that the creation of PCCs have enhanced more or less inter-professional partnership for youth health care services, especially the earlier detection of possible social and health threats. The professionals indicated that multidisciplinary working contributes to an improvement in the continuity of care. However, they also considered PCCs to be a work in progress, especially with regard to the need for uniform multidisciplinary protocols, developing and applying more appropriate ethical, practical and financial structures, privacy regulations and the creation of a patient-centred system of care. This is crucial for the PCC to be able to perform its gatekeeper function between first and secondary health care services and to maintain sustainable, affordable secondary health care services. It is a delicate balance in which PCCs could learn from each other and international experiences such as those in the U.K., Finland and Germany [3, 8, 12].

### Comparison with other studies on inter-professional partnership and collaboration

Our findings are in line with the three groups of characteristics (salient attributes, organizational factors and systemic factors) as reported by Butt et al. [20] that influence the inter-professional partnership. Our results are clustered around five influencing topics (see Table 1): 1. Regular cooperation, 2. The multidisciplinary relations, 3. Working under one roof, 4. Position of the manager, and 5. The future opportunities of PCCs. The first two belong to the *salient attributes;* the other three to the organisational characteristics. Systematic factors such as differences in status of professionals or limited knowledge of professionals were not detected.

In another recent publication Barr [25] stated that structural integration (here the creation of PPCs) in itself is not sufficient to create proper inter-professional cooperation. More is needed, namely actively engaging the workforce as partners in the change process and to educate them in this continuously; Barr deemed this as being of great importance. When comparing such a statement with the findings of the current study it seems that the creation of PCC in Amsterdam was too strongly oriented on structural innovation, e.g., working under one roof, and sufficiently on active engagement and leadership development that would lead to a more dedicated, competent and confident work force of professionals.

Other recent studies show similar results as the current PCC study. For example Goldman et al. [26] also demonstrate the importance of issues that deal with influencing the quality of inter-professional cooperation such as clearly defining professionals’ roles, the scopes of practice, the role of leadership, and having the actual space to practice team-based primary care effectively. Other studies such as those of Holmesland et al. [27] and Kilgore and Lanford [28] mentioned as important factors that influence inter-professional cooperation ‘mutual reliance’ and ‘mutual understanding.’ In the current study these topics were not specifically mentioned by the PCC professionals. Lastly, the model that was developed by Bronstein [29] on the issue of effectiveness of inter-professional processes should be mentioned in light of our PCC findings. He distinguishes five characteristics of these effective processes: 1. Interdependency between professionals; 2. Newly created professional activities; 3. Flexibility between professionals in fulfilling tasks on each other’s domain; 4. Collective ownership of professional goals; and 5. Reflection on the inter-professional processes. Our findings show that characteristic 2 and 3 are less developed in the Amsterdam PCC. The other three are, according to the interviewees, reasonably fulfilled.

### PCCs: a good practice for the Netherlands and elsewhere?

The PCCs have become a leading, positive example for all municipalities in the Netherlands to develop their Youth and Family Centres. They are regarded an improvement to the former system, despite needing further development. After this study, we say this is too much honour for the Amsterdam PCCs. As all interviewees stated, the PCC seem to be a significant structural improvement in the Amsterdam health and social care setting but the inter-professional partnerships could be enhanced when looking at comparable other examples in the literature. Experiences in England, where similar centres have been evolving over the last decade, indicate that such changes will take time [8]. In their 2000 report ‘Team working in primary health care’ the Royal British Pharmaceutical Association and the British Medical Association draw similar conclusions regarding the potential for integrated care in this field, given multidisciplinary efforts and persistence [10]. They also stated that “Effective communication, optimum team size, appropriate autonomy for members of the team and adequate time and resources are also important factors” [10]. Furthermore, in line with our findings, their report also states “Teamwork does not necessarily follow from professionals working alongside one another. Structural, historical and attitudinal barriers can and do contribute to difficulties which inhibit teamwork. Problems can arise from competing demands, diverse lines of management, poor communication, personality factors, plus status and gender effects” [10].

### Limitations of the study

First, as the focus of this study was on the PCCs in Amsterdam, the results may prove difficult to generalize to other multidisciplinary integrated youth health centres elsewhere. However, the PCCs in Amsterdam have from their origin served as a role model for the Youth and Family Centres in the Netherlands. Second, this is a qualitative study which does not show size and frequencies of changes in professional practice. The researchers advise that more quantitative data on the effects of PCCs on professional practices and patient care is needed in future studies to more comprehensively understand the effects of PCCs and PCC-like developments elsewhere. An important strength of our study is its qualitative nature. We investigated the functioning of the PCCs and not their effect on health outcomes of children and parents in comparison to fragmented settings, because that type of research requires Randomized Clinical Trials or a prospective case control design. However, such studies only are useful if the functioning of the new system is stable. This is not (yet) the case regarding Amsterdam’s PCCs: the basic structure is present, but the work processes are still dynamic and not yet finalized. We therefore recommend further outcome studies only once this new system is sufficiently stabilized.

## Conclusion

Youth Health care professionals and their managers perceive advantages and challenges for inter-professional partnership within Parent and Child Centres in comparison to the former system. Several challenges, mostly involving the professional and organizational processes, must be tackled to allow further development of PCCs. Also further research is required to answer the question whether PCCs indeed improve quality of care and child health outcomes.

## Figures and Tables

**Table 1.**
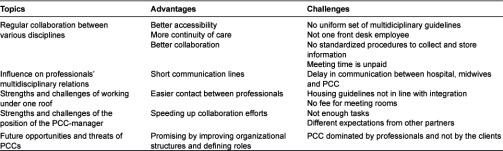
Advantages and challenges per topic of PCCs in comparison to the former, fragmented systems of professionals, providing care to parents and children.
